# Prosthetic Joint Infection due to *Actinomyces* species: A case series and review of literature

**DOI:** 10.7150/jbji.35592

**Published:** 2019-08-02

**Authors:** Ramez Dagher, Talha Riaz, Aaron J. Tande, Douglas R. Osmon, Anil Jagtiani, James M. Steckelberg, Tad Mabry, Elie F. Berbari

**Affiliations:** 1 Department of Internal Medicine and Division of Infectious Diseases, Mayo Clinic College of Medicine, 200 1st Street SW, Rochester, MN 55905.; 2 Department of Orthopedic Surgery, Mayo Clinic College of Medicine, 200 1st Street SW, Rochester, MN 55905.

**Keywords:** prosthetic joint infection, *Actinomyces* spp., two stage exchange, resection arthroplasty

## Abstract

**Background:** *Actinomyces* prosthetic joint infections (APJIs) are rare and optimal medical and surgical treatment strategies are unknown. The purpose of our study was to characterize the demographics, risk factors, management and outcomes of patients with PJIs due to *Actinomyces* spp.

**Methods:** Using a retrospective cohort study design, the medical records of all patients with *Actinomyces* spp. total hip or knee arthroplasty infection (APJI) seen at a single institution between January 1, 1969 and December 31, 2016 were reviewed. We abstracted information including patient demographics, co-morbidities, joint age, surgical history, microbiology, management and outcomes. A simultaneous literature search via PubMed was performed to identify cases of APJI published in literature and a descriptive analysis was performed.

**Results:** Eleven cases were identified over a 47 year study period at our institution. Seven patients (64%) were female. The median age at the time of diagnosis of infection was 71 years (range, 57-89). The knee was involved in six cases (55%) followed by the hip in 5 (45 %) cases. Three cases had dentures, broken teeth, or poor dentition. *Actinomyces odonotlyticus* was the most commonly found subspecies at our institution. Median ESR and CRP values were 61mm/hr and 64 mg/L respectively. Eight (72%) patients were managed with 2 stage exchange. Most patients received a course of beta-lactam therapy for 6 weeks. Ten cases (91%) were free of failure after a median duration of follow-up of 2 years (range, 0.67 - 5 years). The median duration from joint arthroplasty to the onset of symptoms was 162 days, range (20-3318). Six (54%) had a history of prior PJI with a different microorganism at the same joint site and 4 patients had history of prior 2 stage exchange (36%). In the literature group, we identified 12 cases and the most common subspecies was *Actinomyces israelii*; most patients underwent two stage exchange and were treated with 6 weeks of beta lactam antibiotics.

**Conclusions:** Based on our observational study, *Actinomyces* PJI presents as a late complication of TJR, may be associated with prior PJI at the index joint and antecedent dental manipulation may portend as an additional risk factor. Treatment includes two stage exchange and beta- lactam therapy for 6weeks. These results will help clinicians in improved understanding and management of APJIs which although are rare but warrant special attention as population with implanted joint arthroplasties continues to rise.

## Introduction

Anaerobic PJIs are uncommon 1, 2. The most frequent anaerobes reported to cause PJI include *Cutibacterium acnes* and *Bacteriodes* spp 1. PJIs due to *Actinomyces* spp. have been infrequently reported in literature and information regarding their clinical presentation, management and outcome is scarce. In addition the 2012 IDSA guidelines on the management of PJI did not address uncommon anaerobic organisms 3.

*Actinomyces* is a gram-positive facultative anaerobic bacterium and one of the most common commensals of human oral cavity. It is known to colonize the skin, gastrointestinal, respiratory and genitourinary tract 4. Bone and joint infection due to *Actinomyces* spp. including osteomyelitis of the jaw, tibia, foot, sternum, pubic symphysis and vertebral osteomyelitis have been described 4-6.

However, apart from select case reports about APJI, their epidemiology, risk factors, diagnosis and management are not well elucidated. According to one review, APJI infection becomes apparent after 1 year after implantation 2. It has also been reported that the hip is more frequently infected due to its proximity to the gastrointestinal and genitourinary tract 2. Diabetes mellitus was reported to be predisposing risk factor in one case report 7. Intravenous drug abuse and subsequent APJI has also been described 8. There is lack of guidance on the optimal antimicrobial and surgical management of APJI and how it might differ from PJI caused by common microorganisms such as *Staphylococci*, *Streptococci* or other anaerobes. The purpose of this study was to define the demographics, management and outcome of patients with APJIs using a retrospective cohort study design and compare them with the published literature pertaining to APJI. Our aim was to improve our understanding of APJI to help providers in the management of total joint arthroplasty infection.

## Methods

Following institutional review board (IRB) approval, a retrospective study was conducted at a single academic center. The medical records of all episodes of *Actinomyces* spp. total hip or knee arthroplasty infection (APJI) seen at our institution between January 1, 1969 and December 31, 2016 were reviewed. Other joint sites were not included. Cases were identified with the use of the institutional total joint registry. All episodes were followed from the date of the APJI diagnosis until last follow-up or death. Using the MSIS major criteria, APJI was present if at least two separate cultures grew* Actinomyces* spp. from intra-articular surgical tissue specimens or joint aspirations or one positive culture with presence of purulence or sinus tract 9.

Data were collected from the electronic and paper medical record and stored in a password protected database. We abstracted information concerning the patient's demographics, surgical history, clinical presentation, microbiology, laboratory tests, imaging exams, surgical and medical treatment. Data points including date of initial joint arthroplasty, date of most recent surgical intervention on the index joint and date of onset of symptoms were collected. Joint age was defined as the duration of time from last arthroplasty to the date of onset of symptoms. We recorded a history of abdominal, colorectal, oral and uro-gynecologic procedures. Co- morbidities including CKD, DM2, immunosuppression and obesity (BMI > 30) were abstracted. Microbiology and histopathology data was recorded; patients with polymicrobial infection were included. We recorded sub-speciation of* Actinomyces* and * in vitro* susceptibilities (where available). Because of the 47 year study period, microbiologic culture techniques varied over time, however current methodology includes incubating synovial fluid or joint tissue specimens in blood cultures bottles under anaerobic conditions for duration of 14 days at our institution. Synovial fluids must have at least 2 mL to qualify for testing in bottles. If <2 mL, then routine plating process is followed instead. *Actinomyces* spp. are always tested for penicillin and clindamycin susceptibility with additional drugs added per provider's request.

We systematically searched the English-language literature using PubMed as well as secondary sources for case reports pertaining to APJI, using the search terms “*Actinomyces* prosthesis”, “*Actinomyces* prosthetic”, “*Actinomyces* hip”, “*Actinomyces* knee”, “*Actinomyces* arthroplasty” and “*Actinomyces* joint” on PubMed. Twelve case reports of APJI were identified and reviewed 7, 8, 10-17. Likewise, we abstracted demographics, clinical presentation, labs, surgical factors and management data from these case reports. A descriptive analysis was performed among the 2 groups. Median and ranges for demographics, treatment and outcome variables were calculated. However, no formal statistical comparisons were performed.

## Results

### Study population at our institution

Among the eleven cases identified at our institution, 2 cases were diagnosed prior to year 2000. Seven patients (64%) were female. The median age at the time of diagnosis of PJI was 71 years (range, 57-89). The knee was involved in six cases (55%) followed by the hip in 5 (45 %) cases. Six cases (54%) had a history of prior PJI with a different microorganism at same joint site. Six (54%) patients had a prior operation done on the index joint before the diagnosis: two stage exchange and debridement /retention in 4 and 2 patients respectively. The median joint age at the onset of symptoms of APJI was 162 days (range, 20-3318).

The most commonly reported symptom was chronic joint pain (91%). Drainage or sinus tract were observed in 7 patients (63 %). The median ESR was 61 mm/hr (12-137) and median CRP was 64 mg/L (range, 6.7-196.5). Blood cultures were drawn in 7 cases were negative. Other relevant clinical characteristics are compared among the 2 groups in Table 1.

With regards to relevant dental, colonic and uro-gynecological surgical history, two cases reported appendectomy and hernia repair surgeries. One patient had colon surgery three years before the onset of his symptoms (case # 1, Table 2). Another patient had history of prostate cancer necessitating prostatectomy, cystectomy and ileal conduit creation surgery 7 years prior to diagnosis (case #4, Table 2). Receipt of steroids or other immunosuppressive therapy was not observed in the study cohort.

Pre-operative joint aspiration was performed in 4 cases and purulence or blood-tinged fluid was encountered. In these cases, gram stain did not reveal any organisms, although culture grew *Actinomyces spp*. in 2 cases. Of the eleven cases among whom *Actinomyces* was identified, isolates were sub- speciated in 8 cases (Table 2). * In vitro* antimicrobial susceptibility information was available in 10 cases. All strains were penicillin G susceptible (MIC < 0.5 in ten cases) and 5 were resistant to clindamycin (MIC > 256). Susceptibility to ceftriaxone was available in three cases and all 3 were susceptible. Seven strains were resistant to metronidazole. Acute inflammation and granulomatous reaction were the most common histopathological finding in our case series.

In 8 cases, at least one concomitant pathogen was present. Among the co-pathogens, anaerobes such as *Clostridium spp*., *Peptostreptococcus* spp. and *Bacteroides spp.*, as well as *Staphylococci* were among the most commonly encountered pathogen (Table 2).

Information on management and outcomes are summarized for each group in (Table 2 and 3). The median time between resection and re-implantation was 116 days (range, 21 - 264). Resection arthroplasty was performed in 10 cases. Vancomycin, tobramycin or gentamicin beads and polymethylmethacrylate (PMMA) spacers were utilized in 7 out of 10 cases managed with 2 stage exchange. Subsequent re-implantation of prosthesis was performed in 8 out of 10 cases. The median time between resection and re-implantation was 105 days (range, 21 - 260). All patients in our case series received antimicrobial therapy. In eight cases, antimicrobials were administered before the prosthesis was resected. Following resection arthroplasty, a parental beta-lactam based antimicrobial regimen was used for 6 weeks (penicillin G, ceftriaxone or ertapenem) followed by an oral penicillin or doxycycline for an additional 1.5 to 6 months. Presence of concomitant pathogens determined the receipt of additional intravenous antibiotics in the first 6 weeks as well. Six patients received antibiotics following prosthesis re-implantation. One patient was treated until cultures returned negative at 2 weeks; a second patient was treated for 4 weeks post-operatively till the inflammatory markers normalized. Four other subjects were placed on chronic suppression, though the antibiotic treatment in one of those cases (case # 5) was eventually changed to treat another infection and post-operative wound healing complications. Ten cases (91%) were free of failure (lack of signs of radiologic or clinical failure) after a median duration of follow-up of 2 years (range, 0.67 - 5 years). One patient developed wound dehiscence following two stage exchange requiring further irrigation, debridement and spacer placement (3 stage exchange) but developed *Enterococcal* infection of his arthroplasty, eventually necessitating hip disarticulation (case 5).

### Review of cases in literature

The median joint age was 1460 days (range, 60-5840). Eight were female (66 %) and 10 cases (83%) involved the hip (Table 1). Recent dental manipulation was reported among 3 of the APJI cases and one case received prophylactic antibiotics 16.

The most common symptoms were joint pain followed by joint drainage, reported in 11 and 8 cases of the 12 APJI respectively. Other symptoms included erythema, warmth, tenderness, and swelling. X-rays were either normal or showed peri-prosthetic lucency or loose prosthesis (Table 1). Technetium-labeled bone scans in the literature revealed results ranging from normal findings to osteomyelitis and loosening. On histopathological examination, chronic inflammation, foreign body giant cell reaction, calcification, as well as gram-positive rods were among the common findings. However, in one case, no pathological findings were noted 13. On aspiration, pus was typically encountered and gram-positive rods were identified on gram stain in 4 out of 9 reported cases. In 5 cases where gram stain was negative, many leukocytes were seen. Of the 12 APJI cases reported in literature, *Actinomyces israelii* was the most common pathogen (Table 3).Other co-isolates such as *Staphylococcus aureus* and *Staphylococcus hominis* were found in 2 cases [8, 10].

## Discussion

To our knowledge, this is the largest single center case series of APJIs. Our data suggests that most AJPI present as a late complication of PJI and are equally distributed among the hips and knees. Recent dental procedures portend a potential risk for hematogenous seeding; an association between previous PJIs and prior two stage exchange and subsequent risk of APJI was also observed. Two stage revision approach had favorable outcomes.

*Actinomyces* spp., reside mainly in the oral cavity as part of the periodontal plaque flora and also the colon and genitourinary tract of humans. Generally, *Actinomyces israelii* is the most common species reported to cause disease in humans 18 and of the 12 APJI cases reported in literature, *Actinomyces israelii* was identified in 5 cases whereas *Actinomyces odontolyticus* was the most frequently isolated pathogen in our case series. *A. israelii* is more frequently isolated from the gastrointestinal, respiratory and female genital tract 19.

Prosthetic joints can become infected via local trauma (since *Actinomyces* spp. are found in the soil) and hematogenously from endogenous sources. PJIs can be classified into early, delayed and late onset. Whereas early and delayed infections are thought to have been acquired during surgery, late PJIs occur due to hematogenous seeding 20. We observed that APJI presented as a late complication of TJR as seen in most cases at our institution and in literature 16, 17, 21. The late onset presentation may reflect its nature of being an organism of low virulence. While not a lot is known about its virulence properties, the interlinked branched chains of bacilli have been hypothesized to inhibit phagocytosis * in vivo* 22.

Mixed infection in our series was common, as eight cases were poly-microbial. It is to be noted that majority of* Actinomyces*- related infections tend to be polymicrobial 23. It is hypothesized that presence of multispecies leads to host evasion and degradation of host tissue. Co-pathogens act synergistically enhancing the infectious process. Since *Actinomyces* species are frequently isolated from polymicrobial infections, they must be assumed to contribute to the pathogenetic processes involved in these cases [4]. In terms of clinical presentation, actinomycotic PJIs do not differ much from PJIs due to more commonly encountered pathogens such as *Staphylococci* and *Streptococci*. However, joint drainage and sinus tract was more frequently encountered in our case series and the literature group which is not surprising since *Actinomyces* is an indolent pathogen. Likewise, this led to radiologic manifestations such as periprosthetic loosening or lucency (noted more frequently in literature group).

It is well-known that *Actinomyces* species are part of dental biofilm 24, thus clinicians should be aware of this association. We observed that prior periodontal disease and prior dental surgery might lead to theoretical hematogenous dissemination to the prosthetic joints. However, since these were a small proportion of patients in literature group (3/12) and in our case series (1/11) with periodontal disease, the relationship between dental disease or manipulation and risk of APJI remains unclear. Genitourinary and colonic colonization of *Actinomyces* spp. should also be appreciated. Presence of IUD device associated with pelvic actinomycosis in one case led to APJI^25^ and in a second case seen at our institution, an ileal conduit was present. A breach in the mucosal continuity of these surfaces may lead to hematogenous seeding of the joint. If history and physical exam is suggestive of an abdominal source, a CT scan of abdomen and pelvis to characterize the origin of infection is reasonable.

Six patients had history of prior PJI at the index joint in our case series. However, we do not have data in the paper to truly determine an association between prior PJI and subsequent risk of AJPI. This would require further case- control analysis, where as we are merely reporting an observation.

*Actinomyces spp.* is difficult to culture and in the lab, growth is noted within five days and can take up to 15 to 20 days 26. Receipt of prior antibiotics, inadequate short-term incubation period and inhibition of *Actinomyces* due to the presence of co-pathogens leads to a low yield on routine culture. Generally, in all cases of suspected PJIs, 3-5 tissue specimens for anaerobic cultures should be sent and held for up to 14 days. Gram stain typically shows presence of branching filamentous gram positive rods and should always be performed. Molecular techniques such as ribosomal RNA (rRNA) gene sequencing of *Actinomyces* may help with detection. The Matrix-Assisted Laser Desorption Ionization-Time of Flight Mass Spectrometry (MALDI-TOF) can provide rapid and accurate identification of* Actinomyces spp. Actinomyces spp.* are highly beta- lactam susceptible (rarely resistant) as was observed in all cases in our series 18, 26, however, drug susceptibility should always be performed in cases of PJIs as these are considered serious infections.

Two stage exchange was the predominant surgical approach in the majority of patients. Given the chronicity and indolent nature of these infections, prosthesis loosening or bony lucency and abscess formation necessitates the need for resection arthroplasty and staged reconstruction using cemented spacer. This was met with high success with most patients having reasonable outcomes. Use of a vancomycin impregnated spacer is advised following resection arthroplasty as *Actinomyces* spp. are uniformly susceptible to vancomycin 27. Based on our case series, we suggest a minimum 6 weeks course of highly bioavailable oral or parenteral beta lactam antibiotics based on known active or * in vitro* susceptibilities. In cases of beta- lactam intolerance, tetracycline (if susceptible) and vancomycin are reasonable alternatives 27. Long term antimicrobial suppression following 2 stage exchange is controversial and varies on case to case basis. Following re-implantation, 6 weeks to 3 months course of oral amoxicillin or penicillin VK could be considered.

Our study has some weaknesses. Firstly, this is a retrospective study and subjected to variability in terms of data collection. Secondly, due to small number of cases, establishing a true association between risk factors and subsequent development of APJI is not possible.

In conclusion, APJIs present as a late complication of periprosthetic infection. Two stage exchange arthroplasty and beta- lactam antimicrobial therapy for 6 weeks as first-line therapy followed by short term oral suppression is effective. Observations from this study will help provide an understanding regarding the outcomes of APJI and help counsel patients about treatment strategies and outcome.

## Figures and Tables

**Table 1:** Demographic and clinical presentation between the two groups

Variable	Cases at our institution (N= 11)	Cases in Literature (N=12)
Age median years (range)	71 ( 57-89)	66 (36-85)
Female	7 (63%)	8 (66%)
Hips	5	10
knees	6	2
Joint age days (range)	162 (20—3318)	1460 days (60-5840)
CRP ( median and range) mg/L	64 (range 6.7-196.5)^x^	56 (32-123)^x^
Sedimentation rate (median/range) mm/hr	61 (12-137)^x^	76 (45-120)^x^
Prosthesis loosening or bony lucency	8	6
Sinus tract or drainage	7	1
Erythema	2	2
Swelling	5	5
Purulent aspirate	3^x^	8^x^
Gram positive rods on Gram Stain	None^x^	4

^x= where available^

**Table 2:** Cases of APJI at our institution

Cases (our institution)	Joint age( days) at the time of symptom onset	Arthroplasty type	Co- morbidities	* Actinomyces* spp.	Other pathogens	Surgical therapy	Medical therapy	Clinical outcome and follow up
			Relevant past surgeries ( on the index joint)					
1	334	TKA	DM2, BMI >30, gout, smoking, dentures as a child, colon surgery	*Actinomyces odontolyticus*	None	2 stage exchange	Treated with antibiotics but details N/A	Infection cured with good functional outcome
			Prior DAIR due to Staph epidermidis infection 11 months ago					
2	162	THA	Smoking, GERD	*Actinomyces* spp.^x^	Methicillin- resistant *Staphylococcus epidermidis*	2 stage exchange	6 weeks of PO doxycycline	Good functional outcome, no infection recurrence

3	45	TKA	DM2, Anticoagulation	*Actinomyces* spp^x^.	*Peptostreptococcus spp*, *Bacteriodes spp*, *Staphylococcus epidermidis* and *Clostridum ramosum*	2 stage exchange	6 weeks of VANC, CIP and MTZ.	Excellent clinical outcome, well seated prosthesis
			Arthroscopic I and D at index joint due to wound dehiscence					
4	1499	THA		*Actinomyces* spp.^x^	*Actinobaculum schaalii* and *Cutibacterium Acnes*	2 stage exchange	PCN G x 6 wks and PO MTZ x 6 weeks Po AMX 4 weeks post implantation	Well seated THA, good functional outcome
			Prostate Ca s/p Cystprostatectomy, orchiectomy and ileal conduit 6 yrs ago, dental operation 7 months ago					
5	1214	TKA	BMI >30, Smoking	*Actinomyces neuii*	None	2 stage exchange	6 weeks CFTX, 3 months PCN VK long term suppression	*Enterococcal* TKA infection, later left hip dysarticulation due to another infection
			Prior 2 stage exchange due to MSSA TKA infection 3 years ago,					
6	3318	THA	HD dependence	*Actinomyces naeslundii*	None	Girdlestone resection due to poor bone stock	Doxy and Levo x 9 months + IV CFX 2 months, then 4 months of amoxicillin	Resolution of hip pain, but immobile
			*Coxiella burnetti* left THA infection 7 years ago s/p DAIR, on DOX/LEV					
7	1858	TKA	BMI> 30	*Actinomyces europaeus*	*Peptostreptococcus spp*., *MRSA* and *Enterococcus faecalis*	2 stage exchange	6 weeks of VANC and ERT, 6 months of AMX; following reimplantation, indefinite AMX	Well seated components and good functional outcome
			Advanced periodontal disease					
8	152	TKA	GERD, DM2, PAD	*Actinomyces odontolyticus*	*Prevotella melaninogenica,* *Enterococcuspp. , Abiotrophia/Granulicatela spp.,* *Streptococcus viridans, Pandoraea norinbergensis*	Resection arthroplasty, not reimplanted	6 weeks of PCN G and MTZ, 4 weeks TMP/SMX followed by 6 months of AMX	No recurrence of infection, left knee deformity due to removal of the prosthesis and patellectomy
			Prior PJI requiring 2 stage exchange, prior hysterectomy					
9	20	THA	BMI>30	*Actinomyces odontolyticus*	*Peptoniphilus* spp., *Anaerococcus vaginalis*, *Streptococcus agalactiae*, *Trupurella bernardiae and Corynebacterium Striatum*	2 stage exchange	PIP/TAZO and LINEZOLID x 6 wks, then 6 wks of oral PCN	Well seated prosthesis and wires, no recurrence at last follow up
			Prior PJI requiring 2 stage exchange					
10	120	TKA		*Actinomyces odontolyticus*	*Staphylococcus epidermidis*, *Finegoldia magna* and *Enterococcus faecalis*	2 stage exchange	6 weeks of VANC and ERT, following reimplanation, lifelong suppression with AMX/CLAV + DOXY	Pain free, no recurrence
			Prior PJI requiring 2 stage exchange 4 months ago					
11	20	THA	x	*Actinomyces radingae*	Finegoldia magna, Cutibacterium Acnes and Bacillus spp.	DAIR	6 weeks of Ertapenem followed by lifelong PO PCN VK	Pain free, good functionality
			x					

X : not subspeciated, VANC: vancomycin, PCN : Penicillin, CIP: Ciprofloxacin, AMX/CLAV: amoxicillin/clavulanic acid, MTZ: metronidazole, CFX: ceftriaxone, TMP/SMX: trimethoprim/sulfamethoxazole, LEV: levofloxacin, PIP/TAZO: piperacillin/tazobactam, DAIR: debridement, irrigation and retention.

**Table 3:** Cases of APJI in literature

Patients (literature)	Joint age (days)	Location	Previous surgeries/ comorbidities	Actinomyces species/subspecies	Other pathogen	Surgical therapy	Medical therapy	Outcome
1	3650	THA	Steroids, dental extractions and caries filling 1 month before onset of hip pain	*Actinomyces israelii*	No	Resection arthroplasty followed by reimplantation 8 month later	6 weeks of high dose IV Penicillin G	No clinical evidence of infection at 6 month follow up
2	5840	THA	None	*Actinomyces viscosus*	No	Girdlestone resection	6 weeks of IV Penicillin G	Cured, not reimplanted due to poor Bone stock
3	180	THA	None	*Actinomyces naeslundii*	No	Needle aspiration	6 weeks of Rifampin + Cefuroxime	Doing well clinical at last F/U
4	90	TKA	None	*Actinomyces naeslundii*	No	2 stage exchange	9 weeks of IV ceftriaxone and lifelong doxycycline after reimplanation	Good functional outcome, still some pain
5	4015	THA	Steroids, IVDA, femoral head AVN	*Actinomyces israelii*	No	Resection with spacer	Ampicillin IV x 20 days, then left AMA	Lost to follow up
6	730	THA	Dental prosthesis	*Actinomyces neuii subsp. neuii*	No	2 stage exchange	PCN G IV until 2 weeks post reimplantation and then 4 week Amoxicillin 1 gram tid	good radiological control, not seen for follow up
7	3285	THA	DM2	*Actinomyces israelii*	No	2 stage exchange	6 weeks of vancomycin	no clinical or lab evidence of infection
8	60	TKA	DM2, COPD	*Actinomyces naeslundii*	No	2 stage exchange	6 weeks of PCN and clindamycin	no recurrence, doing well
9	300	THA	Dental cleaning without prophylaxis 1 mo ago	*Actinomyces spp*	No	2 stage exchange revision	8 weeks of PCN G, following reimplantation, 1 year of PCN VK	No signs of infection and good functional and radiographic outcome
10	4015	THA	Warfarin therapy, GB syndrome	*Actinomyces gerencseriae*	*Staphylococcus aureus*, *staphylococcus hominis*	I and D	Amoxicillin and TMP/SMX x 3 wks	Cured
11	2190	THA	IUD device with foul smelling discharge	*Actinomyces israelii*	No	Patient refused hip surgery, IUD removed	10 days of PCN G 24 M U/d x 10 days, then lost to F/U	Left AMA
12	365	THA	None	*Actinomyces israelii*	No	Girdlestone resection arthroplasty	High dose IV benzylpenicillin	Superinfection with *Staph aureus* leading to femoral osteomyelitis
